# Mortality risk associated with occupational exposures in people with small airways obstruction

**DOI:** 10.1371/journal.pone.0305125

**Published:** 2024-06-11

**Authors:** Valentina Quintero-Santofimio, Cosetta Minelli, James Potts, Roel Vermeulen, Hans J. Kromhout, Ben Knox-Brown, Johanna Feary, Andre F. S. Amaral

**Affiliations:** 1 National Heart and Lung Institute, Imperial College London, London, United Kingdom; 2 Institute for Risk Assessment Sciences, University of Utrecht, Utrecht, The Netherlands; 3 NIHR Imperial Biomedical Research Centre, London, United Kingdom; Srebrnjak Children’s Hospital, CROATIA

## Abstract

**Background:**

Small airways obstruction (SAO) has been associated with occupational exposures. Whether exposure to harmful occupational agents impacts the survival of people with SAO is unknown. Our aim was to estimate the mortality risk associated with occupational exposures among people with SAO.

**Methods:**

We used data from UK Biobank participants with SAO, defined as a ratio of forced expiratory volume in three seconds to forced expiratory volume in six seconds (FEV_3_/FEV_6_) below the lower limit of normal. We assigned lifetime occupational exposures to participants with available occupational histories using the ALOHA+ Job Exposure Matrix. Mortality data were provided by the National Death Registries. We used Cox regression to assess the association of all-cause mortality with lifetime occupational exposures (vapours, gases, dusts, fumes—VGDF; solvents; pesticides; metals), adjusting for potential confounders.

**Results:**

The 13,942 participants with SAO had a mean age of 56±7 years, 59% were females and 94.2% were of White ancestry. Overall, there were 457 deaths over a median follow-up of 12.8 years. A greater mortality risk was associated with exposure to VGDF, with hazard ratios of 1.32 (95%CI: 1.04–1.78) for low levels and 1.41 (95%CI: 1.11–1.78) for moderate levels of cumulative exposure. There was no evidence of association for the other occupational exposures.

**Conclusion:**

Lifetime occupational exposure to VGDF in people with SAO may have a detrimental effect on their survival. Future respiratory health surveillance programmes of people exposed to VGDF should consider assessment for SAO and focus on primary prevention through adequate exposure control.

## Introduction

Chronic respiratory diseases such as asthma and chronic obstructive pulmonary disease (COPD) are leading causes of morbidity and mortality worldwide. Both asthma and COPD are characterised by airflow obstruction, to which an increase in small airways resistance and obstruction are known contributors [1]. Even when traditional spirometric indices are normal, small airways obstruction (SAO) is common in the general population, and has been associated with occupational exposures to vapours, gases, dusts, and fumes (VGDF), as well as pesticides [2, 3].

Although tobacco smoking is an important risk factor for chronic respiratory diseases, harmful occupational exposures may also contribute to increased mortality among particular groups of people. Two large studies from Sweden have shown that workers exposed to VGDF have an increased risk of mortality [4, 5]. Whether people with SAO are more susceptible to occupational exposures and, as consequence of this, have reduced survival is unknown.

The aim of this study was to assess the association of all-cause mortality with lifetime occupational exposures in UK Biobank participants with spirometrically-defined SAO.

## Methods

### Study population

A total of 502,414 adults, aged 40–69 years, were recruited to UK Biobank between 2006 and 2010 [6]. In this analysis we included participants with SAO at baseline, defined as the ratio between the forced expiratory volume in three seconds and the forced expiratory volume in six seconds (FEV_3_/FEV_6_) less than the lower limit of normal (LLN). This definition was based on findings of our previous study which has shown minimal difference between the forced vital capacity (FVC) and FEV_6_ [7]. We defined isolated SAO as FEV_3_/FEV_6_<LLN in the absence of airflow obstruction (FEV_1_/FEV_6_>LLN). All participants included in this study had high quality spirometry with at least two spirograms, no cough, back extrapolated volume <5% FVC (or >5% but <150mL), reproducible FEV1 and FVC and a forced expiratory time ≥ 6 seconds on the best curve (curve with highest FEV1 and FVC).

Lifetime occupational histories based on job titles for all paid jobs held for longer than six months was collected between July and September 2015 using the Occupational Self-Coding and Automatic Recording tool (OSCAR) [8]. Using the ALOHA + Job Exposure Matrix (JEM), each individual was assigned a cumulative exposure for 10 individual agents and two compound agents, based on intensity and duration of exposure as described in our previous study [7]. Information on the causes of death was obtained via linkage of UK Biobank participants with the death records of the UK Office for National Statistics through National Health Service identification numbers. Death was classified according to its underlying (primary) cause using the International Classification of Diseases, edition 10 (ICD-10). All-cause mortality was defined as death of any cause, except external causes (V01–Y89). The date of latest mortality follow-up was 31 January 2022. Ethical approval was obtained for UK Biobank (National Research Ethics Service, 11/NW/0382, 21/NW/0157). Data was accessed on the 13/09/2022.

### Statistical analysis

The lifetime all-cause mortality risk, from birth to January 2022 (censoring date), associated with lifetime occupational exposures at the time of baseline assessment was determined using a multivariable Cox proportional hazards regression model. This model was adjusted for the following potential confounders: sex, smoking status (never, ex- or current smoker), smoking pack-years, Townsend deprivation index, ethnicity, and UK Biobank assessment centre.

To estimate the hazard ratios (HR) and 95% confidence intervals (CI) for the association between all-cause mortality and occupational exposures in participants with SAO, age instead of time-on-study was used as the time scale [9]. We performed an additional analysis among people with isolated SAO, occupational exposures and all-cause mortality. Analyses were conducted using the *survival* and *survminer* packages in R (version 4.1.1.).

## Results

Included in this analysis were 13,942 (21.4%) UK Biobank participants with lifetime occupational histories available and high-quality spirometry demonstrating SAO at baseline. Of these, 59.4% were females, 51.4% were never smokers, and 94.2% were of white ethnicity. Regarding employment status at the baseline study visit, 61.6% of the participants were in full-time employment and 32.3% were retired. Airflow obstruction, defined as FEV_1_/FEV_6_<LLN, was present in 55.3% of females and 62.1% of males (Table 1). Among participants with SAO, 41.9% (n = 5,847) had isolated SAO defined as FEV_3_/FEV_6_<LLN and FEV_1_/FEV_6_>LLN, and their characteristics were similar to those with SAO.

**Table 1:** Baseline characteristics on the participants with FEV_3_/FEV_6_<LLN in the UK Biobank.

Characteristic	Females	Males	Total
	(n = 8,280)	(n = 5,662)	(n = 13,942)
**Age at recruitment in years, mean (SD)**	55 (7)	58 (8)	56 (7)
**Ethnicity, n (%)**			
**White**	7,764 (93.8%)	5,374 (94.9%)	13,138 (94.2%)
**Non-white**	516 (6.2%)	288 (5.1%)	804 (5.8%)
**Smoking status, n (%)**			
**Never**	4,539 (54.8%)	2,627 (46.4%)	7,166 (51.4%)
**Ex-smoker**	2,923 (35.3%)	2,361 (41.7%)	5,284 (37.9%)
**Current smoker**	818 (9.8%)	674 (11.9%)	1,492 (10.7%)
**Pack-years of smoking, mean (SD)**	6.9 (13.2)	10.3 (18.1)	8.3 (11.3)
**Townsend deprivation index, median (IQR)**	-2.3 (-3.7 to 0.03)	-2.5 (-3.9 to -0.3)	-2.4 (-3.8 to -0.1)
**FEV**_**1**_ **, L, median (IQR)**	2.3 (1.9 to 2.6)	3.0 (2.6 to 3.5)	2.5 (2.1 to 3.0)
**FEV**_**3**_ **, L, median (IQR)**	2.9 (2.5 to 3.3)	3.9 (3.4 to 4.5)	3.2 (2.7 to 3.8)
**FEV**_**6**_ **, L, median (IQR)**	3.2 (2.8 to 3.6)	4.4 (3.8 to 4.9)	3.5 (3.0 to 4.3)
**FEV**_**1/**_**FEV**_**6**_ **<LLN, n (%)**	4,579 (55.3%)	3,516 (62.1%)	8,095 (58.1%)
**Deaths reported, n (%)**	190 (2.3%)	267 (4.7%)	457 (3.3%)
**Cardiovascular deaths, n (%)**	26 (13.7%)	57 (21.3%)	83 (18.2%)
**Respiratory deaths, n (%)**	10 (5.3%)	21 (7.9%)	31 (6.8%)
**Neoplasm death, n (%)**	127 (66.9%)	143 (53.6%)	270 (59.1%)

There were 457 (3.3%) deaths, reported over a median follow-up of 12.8 years. The most common cause of death was neoplasms (59.1%), followed by cardiovascular (18.2%) and respiratory causes (6.8%) (Table 1). More deaths were reported in males (58.4%). Of the total deaths, 66.3% (n = 303) had concurrent airflow obstruction (FEV_1_/FEV_6_<LLN). Among participants with isolated SAO, there were a total of 154 (2.6%) deaths reported over a median of 12.8 years of follow up. Similar to those with SAO, the most common cause of death was neoplasm (55.8%), followed by cardiovascular (22.7%) and respiratory (3.9%). Nearly 50% of participants with SAO were exposed to at least one of the 12 occupational agents of interest, with the commonest exposures being to VGDF (48.5%), all solvents (30.2%), metals (11.5%) and pesticides (4.0%). The highest number of deaths were in participants with occupational exposures to VGDF (58.3%), and fewest deaths were in those exposed to pesticides (5.1%; Table 2).

**Table 2:** Hazard ratios of all-cause mortality for all occupational exposures among participants with FEV_3_/FEV_6_<LLN.

JEM agent (Exposure)	Total				Females				Males			
	N	Deaths (n = 457)	HR (95%CI)	*P*	N	Deaths (n = 190)	HR (95%CI)	*P*	N	Deaths (n = 267)	HR (95%CI)	*P*
**VGDF**												
Low (<2 EU-years)	2,854	110	1.32 (1.04–1.78)	**0.001**	1,798	53	1.52 (1.09–2.13)	**0.01**	1,056	57	1.30 (0.94–1.82)	0.12
Moderate (2–17 EU-years)	3,121	121	1.41 (1.11–1.78)	**0.02**	1,690	38	1.14 (0.78–1.67)	0.5	1,431	83	1.39 (1.03–1.88)	**0.03**
High (≥18 EU-years)	789	35	1.10 (0.76–1.60)	0.6	122	3	1.20 (0.38–3.81)	0.8	667	32	1.07 (0.70–1.61)	0.8
**All pesticides**												
Low (≤1 EU-years)	255	13	1.38 (0.79–2.41)	0.3	76	0	-	-	179	13	1.70 (0.96–3.00)	0.06
Moderate (2–15 EU-years)	49	0	-	-	15	0	-	-	34	0	-	-
High (≥16 EU-years)	240	10	1.26 (0.67–2.36)	0.5	94	3	1.26 (0.40–3.99)	0.7	146	7	1.19 (0.56–2.53)	0.7
**All solvents**												
Low (≤1 EU-years)	2,085	73	1.03 (0.80–1.33)	0.8	1,165	23	0.88 (0.56–1.36)	0.6	920	50	1.10 (0.80–1.52)	0.5
Moderate (2–15 EU-years)	1,289	46	1.05 (0.77–1.43)	0.8	622	14	1.11 (0.64–1.93)	0.7	667	32	0.99 (0.67–1.45)	0.9
High (≥16 EU-years)	842	26	0.83 (0.55–1.23)	0.3	400	9	1.05 (0.53–2.07)	0.9	442	17	0.71 (0.43–1.17)	0.2
**Metals**												
Low (<2 EU-years)	969	51	1.16 (0.85–1.57)	0.4	142	4	1.25 (0.46–3.41)	0.7	827	47	1.16 (0.84–1.60)	0.4
Moderate (2–19 EU-years)	492	25	0.94 (0.62–1.43)	0.8	18	0	-	-	474	25	0.95 (0.62–1.45)	0.8
High (≥20 EU-years)	141	6	0.76 (0.33–1.71)	0.5	2	0	-	-	139	6	0.75 (0.33–1.71)	0.5

Key: JEM, job exposure matrix; VGDF (vapours, gases, dust and fumes); all pesticides (combination of fungicides, insecticides and herbicides); all solvents (combination of aromatic solvents, chlorinated solvents and other solvent.

Exposure to VGDF was associated with higher all-cause mortality risk, with a hazard ratio of 1.32 (95%CI 1.04–1.78) for low cumulative exposure and 1.41 (95%CI 1.11–1.78) for moderate cumulative exposure. However, among females the increased mortality risk was only associated with low cumulative exposure to VGDF with a hazard ratio of 1.52 (95%CI 1.09–2.13), while in males it was only associated with moderate cumulative exposure to VGDF with a hazard ratio of 1.39 (95%CI 1.03–1.88) (Table 2). Among participants with isolated SAO, the hazard ratios for high and moderate exposure to VGDF were not materially different from those for the whole sample. However, these were not statistically significant. There were no statistically significant associations between mortality and any of the other occupational agents.

## Discussion

In this study of people with SAO, a greater all-cause mortality risk was associated with low and moderate lifetime exposure to VGDF. We found no statistical evidence of association of all-cause mortality with high lifetime exposure to VGDF, but it is possible this is due to the small number of deaths in this sub-group of people. Similarly, there was no association between mortality and lifetime occupational exposures among people with isolated SAO.

It has been shown that SAO may be associated with respiratory symptoms early on [10, 11], and that it may predict chronic airflow obstruction (as in COPD) later in life [10, 12]. It is plausible that this process may be additionally aggravated by occupational exposures. For example, in a longitudinal study of construction workers in Sweden, exposure to VGDF was associated with increased all-cause mortality and mortality from COPD [4]. Our findings suggest that individuals with SAO may be more susceptible to the negative effects of occupational VGDF due to their predisposition to develop obstructive lung disease over time [13]. It is conceivable that VGDF in the workplace may exacerbate the obstruction of the small airways, leading to faster lung function decline and ultimately impacting survival. However, the mechanism by which occupational exposures may cause increase mortality in those with isolated SAO needs further research. Primary prevention of disease through adequate control of exposure to VGDF in workplaces should be continually reviewed and changes implemented to provide a safer work environment. This includes carrying out air quality monitoring and implementing engineering controls such as ventilation systems, and if necessary, use of appropriate personal protective equipment. We observed no associations with other metal or solvents, however it is plausible that exposure to these agents may occur through alternative routes to inhalation, such as dermal contact, and may have a lesser impact on respiratory health.

The strengths of this study are its large sample size, the use of a standardised protocol for spirometry and lifetime occupational history. However, the UK Biobank is not representative of the UK population and has a healthy volunteer bias, meaning that the findings of this study may not be generalisable to other populations. Whilst there is lack of agreement about how best to define SAO using spirometric parameters, we recently reported FEV_3_/FEV_6_<LLN to be the best parameter in the UK Biobank [7]. This is supported by the findings of a recent study showing a correlation between FEV_3_/FEV_6_-defined SAO and SAO defined by gas trapping and chest computed tomography [10, 14].

## Conclusion

Lifetime occupational exposure to VGDF may contribute to increased all-cause mortality in people with SAO. Our study highlights the need for primary prevention of disease through adequate control of exposures and to consider early monitoring of SAO on workers exposed to VGDF, and primary prevention through adequate exposure control as part of respiratory health surveillance. This will aid to identify and better manage those at risk of respiratory morbidity and mortality in the workplace.

## Supporting information

S1 Table Baseline characteristics on the participants with isolated FEV_3_/FEV_6_<LLN in the UK Biobank. (DOCX)

S2 Table Hazard ratios of all-cause mortality for all occupational exposures among participants with isolated FEV_3_/FEV_6_<LLN. (DOCX)

S1 Fig Directed acyclic graph (DAG) of relationship between occupational exposures in people with SAO and all-cause mortality. (TIF)

S2 Fig Sex distribution of exposures to 12 occupational agents among UK Biobank participants with FEV_3_/FEV_6_<LLN. (TIF)
