# Supplementary material for: Mortality risk associated with occupational exposures in people with small airways obstruction
Source: PLoS One. 2024 Jun 11;19(6):e0305125. doi: 10.1371/journal.pone.0305125 (PMC11166274; doi:10.1371/journal.pone.0305125)
Supplement: S1 Table — (DOCX) [file pone.0305125.s001.docx]

**Mortality risk associated with occupational exposures in people with small airways obstruction**

**V Quintero-Santofimio^1^, C Minelli^1^, J Potts^1^, R Vermeulen^2^, H Kromhout^2^, B Knox-Brown^1^, J Feary^1,3*^, AFS Amaral^1,3*^**

**Supplementary information**

**Table S1**. Baseline characteristics on the participants with isolated FEV_3_/FEV_6_<LLN in the UK Biobank.

| **Characteristic** | **Females**  **(n = 3,505)** | **Males**  **(n = 2,342)** | **Total**  **(n = 5,847)** |
| --- | --- | --- | --- |
| **Age at recruitment in years, mean (SD)** | 56 (7) | 58 (8) | 56 (8) |
| **Ethnicity, n (%)** |  |  |  |
| **White** | 3,258 (92.9%) | 2,206 (94.2%) | 5,464 (93.4%) |
| **Non-white** | 247 (7.1%) | 135 (5.8%) | 382 (6.6%) |
| **Smoking status, n (%)** |  |  |  |
| **Never** | 2,226 (63.5%) | 1,240 (52.9%) | 3,466 (59.2%) |
| **Ex-smoker** | 1,095 (31.6%) | 918 (39.3%) | 2,013 (34.4%) |
| **Current smoker** | 184 (4.9%) | 184 (7.8%) | 368 (6.4%) |
| **Pack-years of smoking, mean (SD)** | 3.9 (9.5) | 7.2 (14.5) | 5.2 (11.9) |
| **Townsend deprivation index, median (IQR)** | -2.5 (-3.8 to -0.4) | -2.6 (-3.9 to -0.5) | -2.5 (-3.8 to -0.4) |
| **FEV_1_, L, median (IQR)** | 2.4 (2.1 to 2.7) | 3.3 (2.9 to 3.7) | 2.7 (2.3 to 3.2) |
| **FEV_3_, L, median (IQR)** | 2.9 (2.5 to 3.3) | 3.9 (3.5 to 4.5) | 3.2 (2.7 to 3.8) |
| **FEV_6_, L, median (IQR)** | 3.2 (2.8 to 3.6) | 4.4 (3.8 to 4.9) | 3.5 (3.0 to 4.3) |
| **Deaths reported, n (%)** | 63 (1.8%) | 91 (3.9%) | 154 (2.6%) |
| **Cardiovascular deaths, n (%)** | 10 (15.9%) | 25 (27.5%) | 35 (22.7%) |
| **Respiratory deaths, n (%)** | 1 (1.6%) | 5 (5.5%) | 6 (3.9%) |
| **Neoplasm death, n (%)** | 40 (63.5%) | 46 (50.5%) | 86 (55.8%) |
