# Supplementary material for: Mortality risk associated with occupational exposures in people with small airways obstruction
Source: PLoS One. 2024 Jun 11;19(6):e0305125. doi: 10.1371/journal.pone.0305125 (PMC11166274; doi:10.1371/journal.pone.0305125)
Supplement: S2 Table — (DOCX) [file pone.0305125.s002.docx]

**Table S2.** Hazard ratios of all-cause mortality for all occupational exposures among participants with isolated FEV_3_/FEV_6_<LLN

| **JEM agent (Exposure)** | **Total** | | | | **Females** | | | | **Males** | | | |
| --- | --- | --- | --- | --- | --- | --- | --- | --- | --- | --- | --- | --- |
|  | **N** | **Deaths (n=154)** | **HR (95%CI)** | ***P*** | **N** | **Deaths (n=63)** | **HR (95%CI)** | ***P*** | **N** | **Deaths (n=91)** | **HR (95%CI)** | ***P*** |
| **VGDF** |  |  |  |  |  |  |  |  |  |  |  |  |
| Low (<2 EU-years) | 1,217 | 35 | 1.27 (0.84-1.90) | 0.3 | 825 | 12 | 0.93 (0.48-1.79) | 0.8 | 392 | 23 | 1.59 (0.93-2.72) | 0.08 |
| Moderate  (2-17 EU-years) | 1,212 | 40 | 1.38 (0.93-2.05) | 0.11 | 698 | 13 | 1.30 (0.68-2.47) | 0.4 | 514 | 27 | 1.55 (0.98-2.59) | 0.10 |
| High (≥18 EU-years) | 305 | 6 | 0.64 (0.27-1.49) | 0.3 | 56 | 1 | 1.05 (0.14- 7.83) | 0.9 | 249 | 5 | 0.58 (0.22-1.51) | 0.3 |
| **All pesticides** |  |  |  |  |  |  |  |  |  |  |  |  |
| Low (≤1 EU-years) | 104 | 4 | 1.43 (0.52-3.95) | 0.5 | 31 | 0 | - | - | 73 | 4 | 1.40 (0.50-3.95) | 0.5 |
| Moderate  (2-15 EU-years) | 17 | 0 | - | - | 5 | 0 | - | - | 12 | 0 | - | - |
| High (≥16 EU-years) | 77 | 3 | 1.17 (0.37-3.68) | 0.8 | 34 | 1 | 1.81 (0.24- 13.6) | 0.9 | 43 | 2 | 0.99 (0.24-4.06) | 0.9 |
| **All solvents** |  |  |  |  |  |  |  |  |  |  |  |  |
| Low (≤1 EU-years) | 840 | 22 | 0.99 (0.62- 1.57) | 0.9 | 516 | 4 | 0.56 (0.20-1.56) | 0.3 | 324 | 18 | 1.22 (0.71-2.08) | 0.5 |
| Moderate  (2-15 EU-years) | 516 | 12 | 1.05 (0.47-1.57) | 0.6 | 257 | 3 | 0.88 (0.27-2.87) | 0.8 | 259 | 9 | 0.84 (0.41-1.72) | 0.6 |
| High (≥16 EU-years) | 332 | 10 | 0.83 (0.49-1.82) | 0.9 | 177 | 6 | 1.76 (0.74-4.19) | 0.2 | 155 | 4 | 0.54 (0.19-1.52) | 0.2 |
| **Metals** |  |  |  |  |  |  |  |  |  |  |  |  |
| Low (<2 EU-years) | 362 | 12 | 0.75 (0.41-1.39) | 0.4 | 67 | 1 | 1.26 (0.17-9.27) | 0.8 | 295 | 11 | 0.73 (0.39-1.40) | 0.3 |
| Moderate  (2-19 EU-years) | 175 | 6 | 0.75 (0.33-1.75) | 0.5 | 11 | 0 | - | - | 164 | 6 | 0.78 (0.33-1.84) | 0.6 |
| High (≥20 EU-years) | 54 | 2 | 0.75 (0.18-3.00) | 0.7 | 2 | 0 | - | - | 52 | 2 | 0.72 (0.17-3.00) | 0.7 |
