# Supplementary figures and images for: Mortality risk associated with occupational exposures in people with small airways obstruction
Source: PLoS One. 2024 Jun 11;19(6):e0305125. doi: 10.1371/journal.pone.0305125 (PMC11166274; doi:10.1371/journal.pone.0305125)

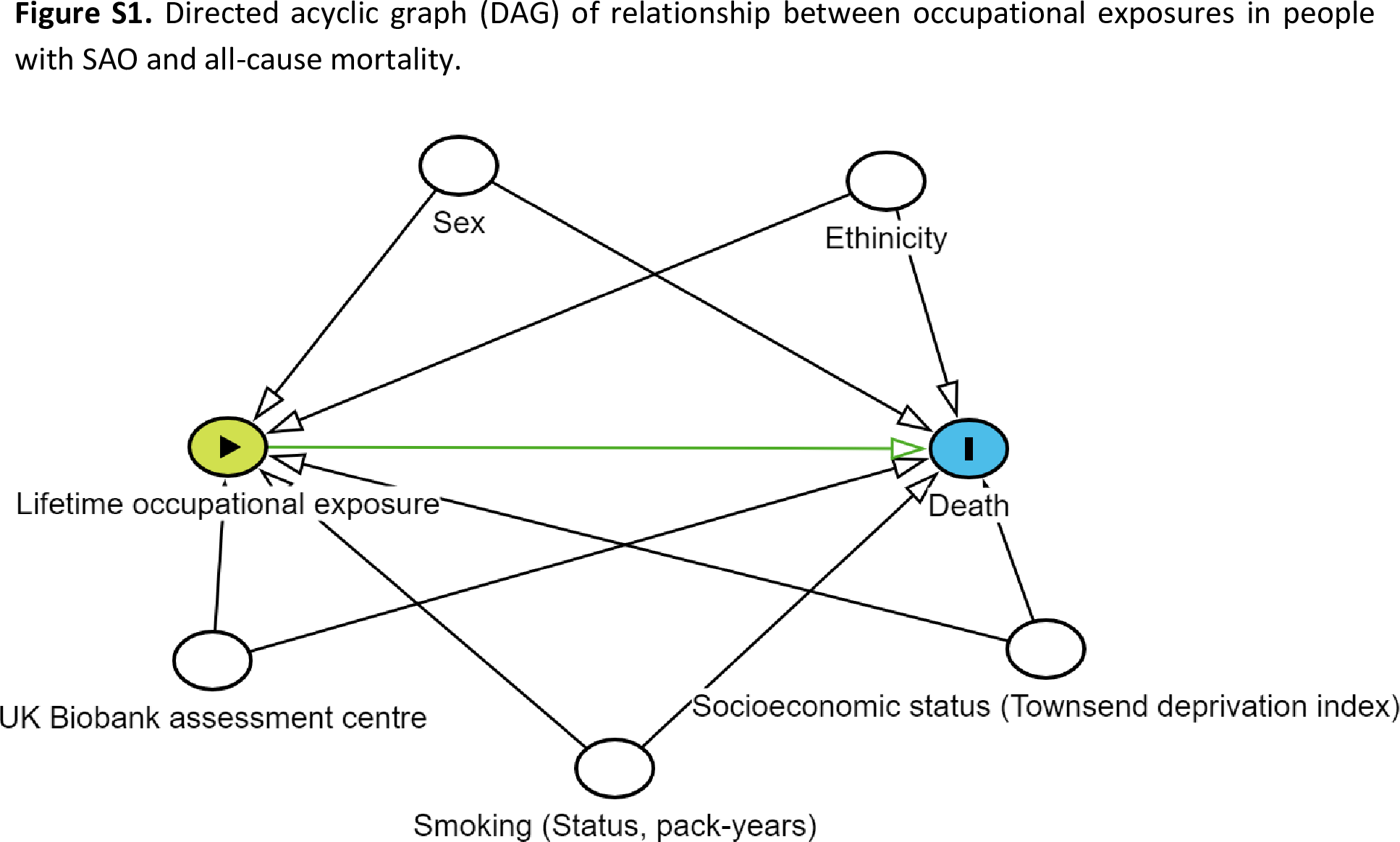

Supplement: S1 Fig — (TIF) [file pone.0305125.s003.tif]

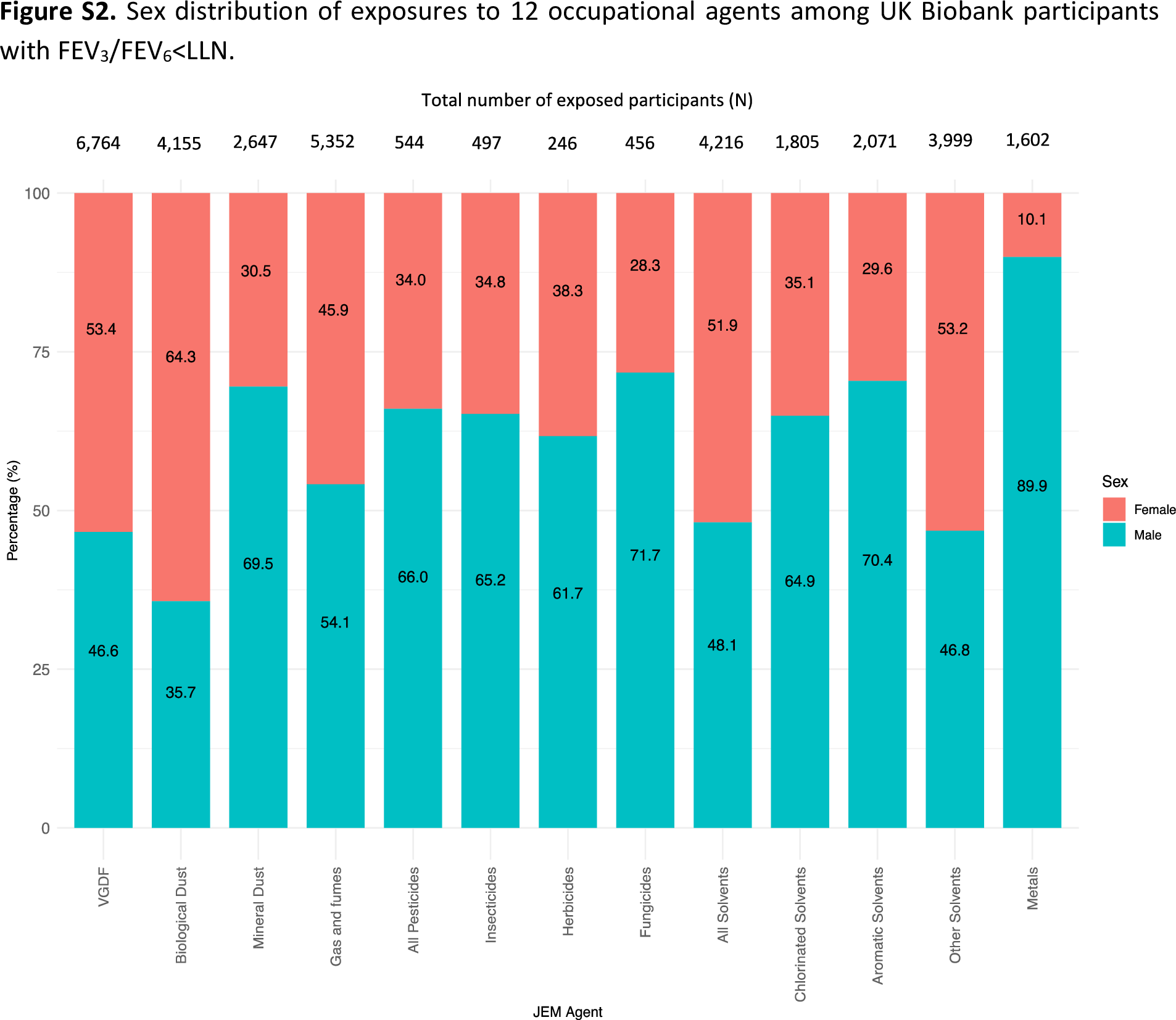

Supplement: S2 Fig — (TIF) [file pone.0305125.s004.tif]
